# Comparison of Dietary Oils with Different Polyunsaturated Fatty Acid n-3 and n-6 Content in the Rat Model of Cutaneous Wound Healing

**DOI:** 10.3390/ijms21217911

**Published:** 2020-10-24

**Authors:** Tomas Komprda, Zbysek Sladek, Zuzana Sevcikova, Veronika Svehlova, Jan Wijacki, Roman Guran, Tomas Do, Zuzana Lackova, Hana Polanska, Lucie Vrlikova, Vendula Popelkova, Petr Michalek, Ondrej Zitka, Marcela Buchtova

**Affiliations:** 1 Department of Food Technology, Mendel University in Brno, Zemedelska 1, 61300 Brno, Czech Republic; tomas.komprda@mendelu.cz (T.K.); xrozikov@mendelu.cz (V.S.); xpopelk8@node.mendelu.cz (V.P.); 2 Department of Animal Morphology, Physiology and Genetics, Mendel University in Brno, Zemedelska 1, 61300 Brno, Czech Republic; zbysek.sladek@mendelu.cz (Z.S.); jan.wijacki@mendelu.cz (J.W.); 3 Laboratory of Molecular Morphogenesis, Institute of Animal Physiology and Genetics, v.v.i., Czech Academy of Sciences, Brno, Veveri 97, 60200 Brno, Czech Republic; sevcikova618@gmail.com (Z.S.); vrlikova@iach.cz (L.V.); 4 Department of Chemistry and Biochemistry, Mendel University in Brno, Zemedelska 1, 61300 Brno, Czech Republic; r.guran@email.cz (R.G.); DoTomas@seznam.cz (T.D.); zuzana.lackova@mendelu.cz (Z.L.); petr.michalek@mendelu.cz (P.M.); zitkao@seznam.cz (O.Z.); 5 Central European Institute of Technology, Brno University of Technology, Purkynova 123, 61200 Brno, Czech Republic; 6 Department of Pathological Physiology, Faculty of Medicine, Masaryk University, Kamenice 5, 62500 Brno, Czech Republic; hana.polanska@gmail.com; 7 Section of Animal Physiology and Immunology, Department of Experimental Biology, Faculty of Science, Masaryk University, 62500 Brno, Czech Republic

**Keywords:** polyunsaturated fatty acids, hydroxyproline, collagen I/III, MPO, macrophages, mast cells, matrix-assisted-laser-desorption-ionization-mass-spectrometry-imaging

## Abstract

Dietary supplementation with polyunsaturated fatty acids (PUFA) n-3 can affect cutaneous wound healing; however, recent findings demonstrate the variable extent of their influence on the quality of healing. Here, we compare the effect of several dietary oils, containing different levels of PUFA n-3 and PUFA n-6, on wound healing in the rat model. Rats were fed the feed mixture with 8% palm oil (P), safflower oil (S), fish oil (F) or *Schizochytrium* microalga extract (Sch) and compared to the animals fed by control feed mixture (C). Dorsal full-thickness cutaneous excisions were performed after 52 days of feeding and skin was left to heal for an additional 12 days. Histopathological analysis of skin wounds was performed, including immune cells immunolabeling and the determination of hydroxyproline amount as well as gene expression analyses of molecules contributing to different steps of the healing. Matrix-assisted-laser-desorption-ionization mass-spectrometry-imaging (MALDI-MSI) was used to determine the amount of collagen α-1(III) chain fragment in healing samples. Treatment by *Schizochytrium* extract resulted in decrease in the total wound area, in contrast to the safflower oil group where the size of the wound was larger when comparing to control animals. Diet with *Schizochytrium* extract and safflower oils displayed a tendency to increase the number of new vessels. The number of MPO-positive cells was diminished following any of oil treatment in comparison to the control, but their highest amount was found in animals with a fish oil diet. On the other hand, the number of CD68-positive macrophages was increased, with the most significant enhancement in the fish oil and safflower oil group. Hydroxyproline concentration was the highest in the safflower oil group but it was also enhanced in all other analyzed treatments in comparison to the control. MALDI-MSI signal intensity of a collagen III fragment decreased in the sequence C > S > Sch > P > F treatment. In conclusion, we observed differences in tissue response during healing between dietary oils, with the activation of inflammation observed following the treatment with oil containing high eicosapentaenoic acid (EPA) level (fish oil) and enhanced healing features were induced by the diet with high content of docosahexaenoic acid (DHA, *Schizochytrium* extract).

## 1. Introduction

Wound healing is a dynamic process aiming to restore the structure of the injured tissue [1], which is characteristic by sequential events progressing through several stages including the inflammation, cell proliferation and migration, angiogenesis, synthesis of the provisional matrix, collagen deposition, and re-epithelialization [1,2,3].

One possibility of how to enhance wound healing is a dietary administration of oils with high content of polyunsaturated fatty acids (PUFA), especially long-chain (LC) PUFA n-3: eicosapentaenoic acid (EPA, 20:5 n-3) and docosahexaenoic acid (DHA, 22:6 n-3). EPA/DHA have anti-inflammatory properties, based, among others, on the competition with PUFA n-6 arachidonic acid (AA; produced from linoleic acid, 18:2 n-6) in eicosanoid synthesis [4] and on the modulation of signaling pathways mediated by transcription factors such as PPARα, PPARγ and NF-κB [5].

EPA and DHA are endogenous ligands of PPARγ, whose activation increases the amount of the adipose tissue-derived anti-inflammatory hormone adiponectin [6]. The EPA/DHA anti-inflammatory effect is further mediated by GPR120, a G-protein coupled receptor, whose activation leads to a repression of the macrophage-induced inflammation [7]). EPA/DHA decreases plasma levels of pro-inflammatory markers interleukin 6 (IL-6), tumor necrosis factor alpha (TNF-α) or interferon γ (IFN-γ), and increases the concentration of anti-inflammatory markers IL-10 and transforming growth factor beta (TGF-β) [8]. 

PUFA n-3-derived lipid mediators play pivotal roles in the initiation of inflammation (prostaglandins and leukotrienes), but on the other hand, they promote and stimulate the active resolution of inflammation (resolvins and protectins) [9,10]. These molecules are therefore able to avert excessive accumulation of neutrophils, which are the first to arrive at the site of inflammation within the tissue, and they can cause tissue damage and amplification of the inflammatory response [11]. Furthermore, in in vitro experiments, EPA and DHA decreased lipopolysaccharide (LPS)-induced pro-inflammatory IL-1β expression in 3T3-L1 adipocytes [12,13] and DHA decreased the level of monocyte chemoattractant protein-1 and IL-6 secreted by murine adipocytes [14]. From the above-mentioned facts, it can be concluded that the PUFA n-3 (their metabolites) are able to modulate especially initial phases of wound healing [15].

However, LC-PUFA n-3 dietary administration in animal models of wound healing provides to some extent the inconsistent results: lower/higher counts of the inflammatory cells in the healing tissue; decreased/increased concentration of both the pro- and anti-inflammatory cytokines; decreased/increased collagen deposition; accelerated/delayed wound healing process [16]. For example, dietary fish oil (LC-PUFA n-3) was previously presented to impair cutaneous wound healing in rats in comparison with sunflower oil (PUFA n-6) treatment [17]. Fish oil in this case caused higher abundancy of inflammatory cells, higher density of dilated blood vessels, and higher density of collagen fibers in cutaneous wounds [17]. Fish oil was also assessed as less suitable in comparison to olive oil during the healing of excisional lesions in mice due to reduced wound contraction, slower re-epithelialization, lower hydroxyproline level, and blood vessels density [18]. Higher intensity of inflammation (in comparison with the control) was also observed in the DHA-fed group of rats that underwent restorative proctocolectomy [19]. On the other hand, DHA exhibited anti-oxidative, anti-inflammatory and anti-fibrotic characteristics due to its ability to reduce TGF-β1 (including its fibrogenic potential), IL-1β and collagen expression, and to decrease the α-SMA (smooth muscle actin)-positive matrix in cholestatic liver injury in rats [20]. Similarly, dietary supplementation with PUFA n-3 (in combination with ascorbic acid) improved healing of ischemic colonic anastomoses in rats [21]. DHA also protected and functionally enhanced repair of the spinal cord after its injury in rats [22]. Moreover, DHA decreased inflammation and joint destruction in mice with the collagen-induced arthritis [23]. 

Taking into account the above-mentioned inconsistences, the aim of our study was to test how different dietary oils enriched with EPA and DHA will affect individual parameters of the skin healing process (wound extent, collagen production/ maturation, angiogenesis, and inflammation) in the rat model following the cutaneous excision and how these effects will contrast to the treatment with diet containing high level of PUFA n-6.

## 2. Results

### 2.1. EPA/DHA Content in the Mebolically Active Adipose Tissue Was Affected by Fish Oil and Schizochytrium Extract Diet

**Figure 1:**
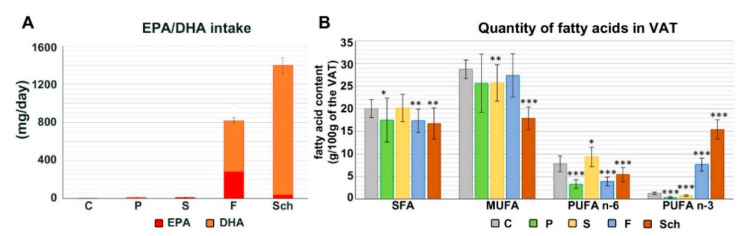
Fatty acid intake and deposition of fatty acid in target tissue. (**A**): EPA/DHA daily intake (EPA in red, DHA in orange) from individual diets: a standard diet alone (control, C), or a standard diet enriched with 8% of palm oil (P), safflower oil (S), fish oil (F) and *Schizochytrium* extract (Sch). (**B**): Fatty acid deposition in the visceral adipose tissue (VAT). Stars labeled group of fatty acids differ significantly from the control group (* *p* < 0.05; ** 0.01 < *p* < 0.05; *** *p* < 0.001; unpaired *t*-test; *n* = 10), average values and standard deviations are displayed in these graphs.

The rats were fed for 64 days either the control (C) basic feed mixture or the basic feed mixture supplemented with 8% of fish oil (F; important source of EPA), 8% of oil extracted from the *Schizochytrium* microalga (Sch; high DHA content), 8% of safflower oil (S; high content of PUFA n-6 linoleic acid), and 8% of palm oil (P; rich in saturated palmitic acid) (Figure 1A; Figure S1A,B). Content of PUFA n-3 in the visceral adipose tissue was significantly increased in the animals fed with fish oil and *Schizochytrium* extract (Figure 1B) and these differences were significant in comparison to control (*p* < 0.05). Any of experimental diet did not significantly affected the weight of the rats (Figure S2A). Only dietary safflower oil decreased daily weight gain of rats in comparison with *Schizochytrium* extract (4.04 vs. 4.56 g/day) (Figure S2B).

### 2.2. Wound Size Was Reduced in Schizochytrium Extract Treated Animals

**Figure 2:**
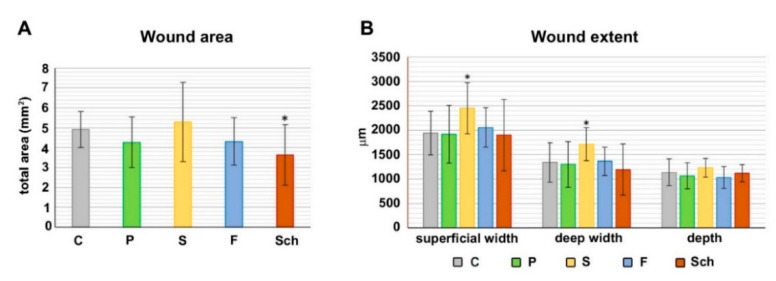
Extent of the wound was measured on transversal histological sections stained by Hematoxylin-Eosin. Control (C), palm oil (P), safflower oil (S), fish oil (F) and *Schizochytrium* extract (Sch) treated animals were analyzed 12 days after skin excision. (**A**): Total wound area. (**B**): Wound extents at different aspects. * labeled group of fatty acids differ significantly from the control group (*p* < 0.05; unpaired *t*-test; *n* = 10), average values and standard deviations are displayed in both graphs.

**Figure 3:**
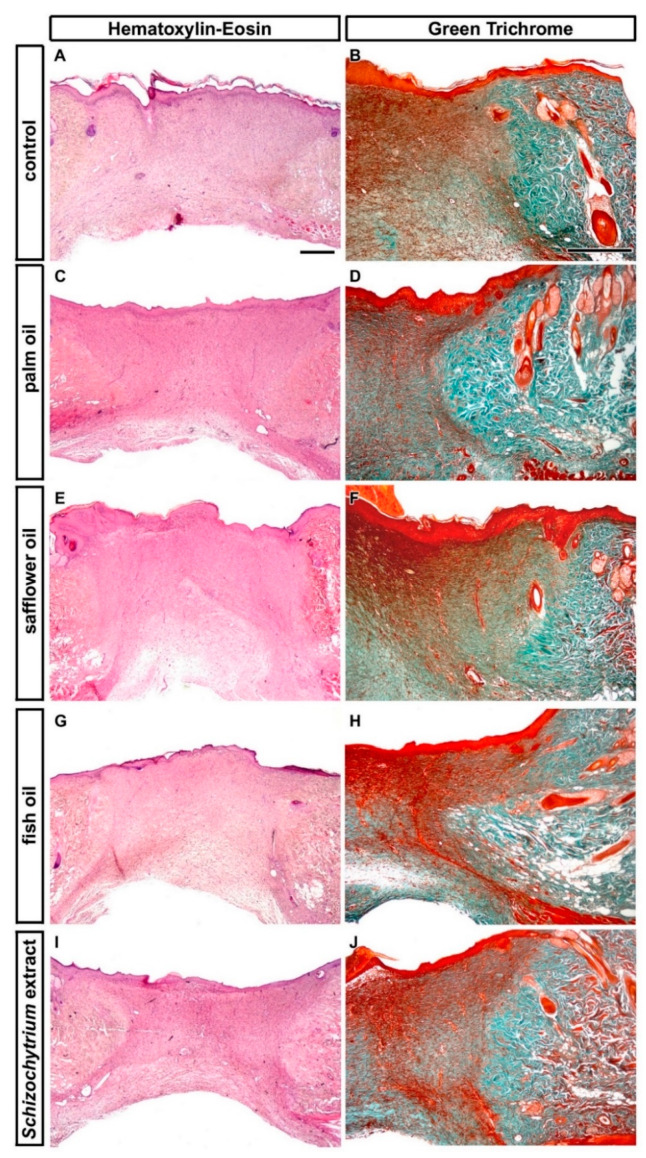
Microscopic structure of wound area. Transversal sections through the wound at 12 days post-excision of rats fed 52 days a standard diet alone (control) (**A**,**B**), or a standard diet enriched with palm oil (**C**,**D**), safflower oil (**E**,**F**), fish oil (**G**,**H**), and *Schizochytrium* extract (**I**,**J**), respectively, stained by Hematoxylin-Eosin (first column) and Masson’s Green Trichrome (second column). Scale bar = 100 µm.

The excision of skin was performed after 52 days of feeding with individual diets and then animals were retained on these diets for an additional 12 days post-excision to analyze their effect on skin healing (in total 64 days of feeding by individual diets). Collected skin samples were first evaluated by common histopathological analyses using Hematoxylin-Eosin staining to determine an area of the cutaneous wound (Figure 2 and Figure 3). Masson’s Green Trichrome staining was further used to visualize the wound extent and character of connective tissues during wound healing where mature collagen fibers are stained in green and immature fibers in red in the healing wound (Figure 3). Dietary palm oil, fish oil, and *Schizochytrium* extract diminished the total wound area as well as other healing parameters (wound width in superficial and deep area; depth of the wound) in comparison with control (Figure 2). The total wound area was significantly smaller (*p* < 0.05) in Sch group in comparison to control (Figure 2A). On the other hand, dietary safflower oil slightly increased all parameters of the wound extent as well as total wound size; however, only wound width in the superficial area was statistically significantly larger in comparison to control (*p* < 0.05) (Figure 2B; all analyzed areas are shown in Figure 14B). 

### 2.3. Angiogenesis Was Enhanced after Schizochytrium Extract Treatment

**Figure 4:**
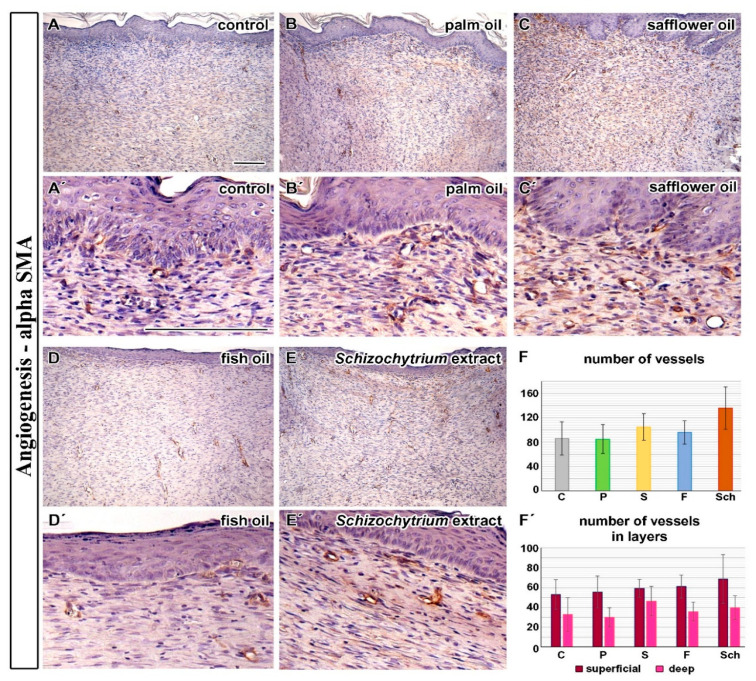
Immunohistochemical labeling of vessels by alpha-SMA. (**A**–**E**,**A’**–**E’**): Vessels were visualized by immunohistochemical labeling of alpha-SMA in individual treatments: control (C), palm oil (P), safflower oil (S), fish oil (F), and *Schizochytrium* extract (Sch) treated animals were analyzed 12 days after skin excision. (**F**): The number of vessels quantified on 10 histological sections for each treatment. (**F’**): The number of vessels separately in the superficial and deep layer of the skin based on immunohistochemical labeling of alpha-SMA. Average values and standard deviations are displayed in both graphs. Differences were not statistically significant (unpaired *t*-test; *n* = 10). Scale bar = 100 µm.

To analyze angiogenesis during wound healing, we first detected blood vessels by the alpha-SMA immunohistochemical staining (Figure 4A–E) and counted their number in individual wounds (Figure 4F,F’). All experimental dietary oils increased the number of blood vessels in the cutaneous wound in comparison with the control diet (Figure 4F), with the highest increase observed after the *Schizochytrium* extract and safflower oil treatment; however, the results were not statistically significant. The proportion of new blood vessels found in the superficial and deep layers of the dermis was almost the same in the control and most of experimental samples, except for the *Schizochytrium* extract where the proportion of blood vessels was slightly increased in the superficial layer of the dermis and safflower oil, where their number was increased in the deep layer of the dermis (Figure 4F’). 

**Figure 5:**
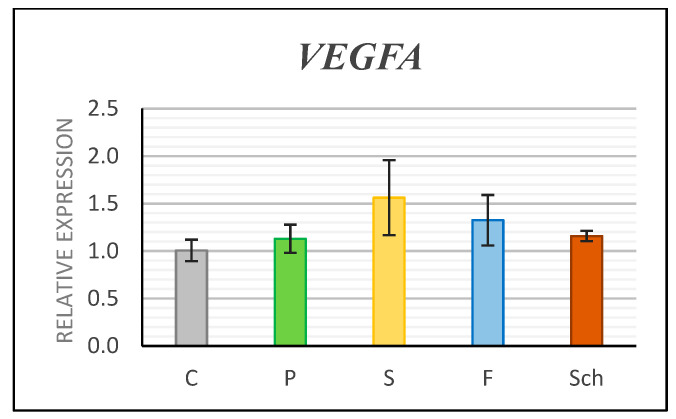
Gene expression of *VEGFA* as analyzed by QPCR. Control (C), palm oil (P), safflower oil (S), fish oil (F), and *Schizochytrium* extract (Sch) treated animals were analyzed 12 days after skin excision. Average values and standard deviations are displayed in the graph.

Next, we analyzed the gene expression of angiogenesis marker *Vascular Endothelial Growth Factor A* (*VEGFA*). The expression of the *VEGFA* was insignificantly upregulated by all dietary oils, but the safflower group exhibited the highest increase in RNA level (Figure 5) in comparison to controls. It is necessary to mention that samples for expression analyses were collected only from a small proportion of skin and therefore this result corresponds to increased number of vessels observed in alpha-SMA labeled samples in deeper layers of treated animals (Figure 4F’). 

### 2.4. Fish Oil Increased Inflammatory Cells Infiltrate 

Inflammatory cells play a key role in several processes during wound healing. They can secrete chemokines or cytokines, release lysosomal enzymes and reactive oxygen species, or contribute to removal of cell debris. Here, we first focused on the detection of neutrophils, macrophages, and mast cells in a wound. 

**Figure 6:**
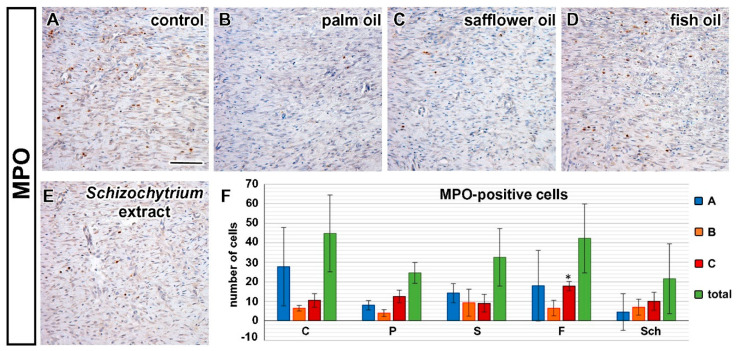
Detection of MPO-positive cells detected by immunohistochemistry. Control (C), palm oil (P), safflower oil (S), fish oil (F), and *Schizochytrium* extract (Sch) treated animals were analyzed 12 days after skin excision. (**A**–**E**): Photos were taken in the middle (“B”) layer (see for explanation Figure 14C); (**F**): Number of MPO-positive cells in the superficial layer (A, blue), in the middle (B, orange) and in the deep layer of the dermis (C, red). Sum of positive cells (green, total). Average values and standard deviations are displayed in the graph. (* *p* < 0.05; unpaired *t*-test; *n* = 5). Scale bar = 100 µm.

**Figure 7:**
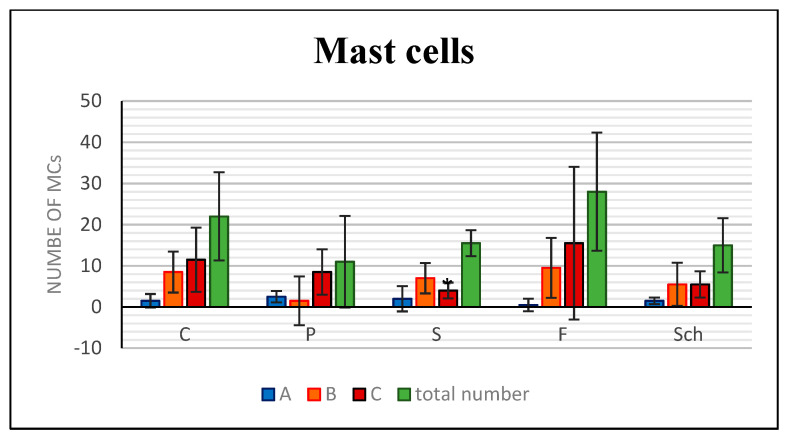
Analysis of mast cells (MCs) number based on their labeling by Toluidine Blue. Control (C), palm oil (P), safflower oil (S), fish oil (F), and *Schizochytrium* extract (Sch) treated animals were analyzed 12 days after skin excision. Number of mast cells in the superficial layer (A, blue), in the middle (B, orange) and in the deep layer of the dermis (C, red). Sum of positive cells (green, total). Average values and standard deviations are displayed in the graph. (* *p* < 0.05; unpaired *t*-test; *n* = 5).

**Figure 8:**
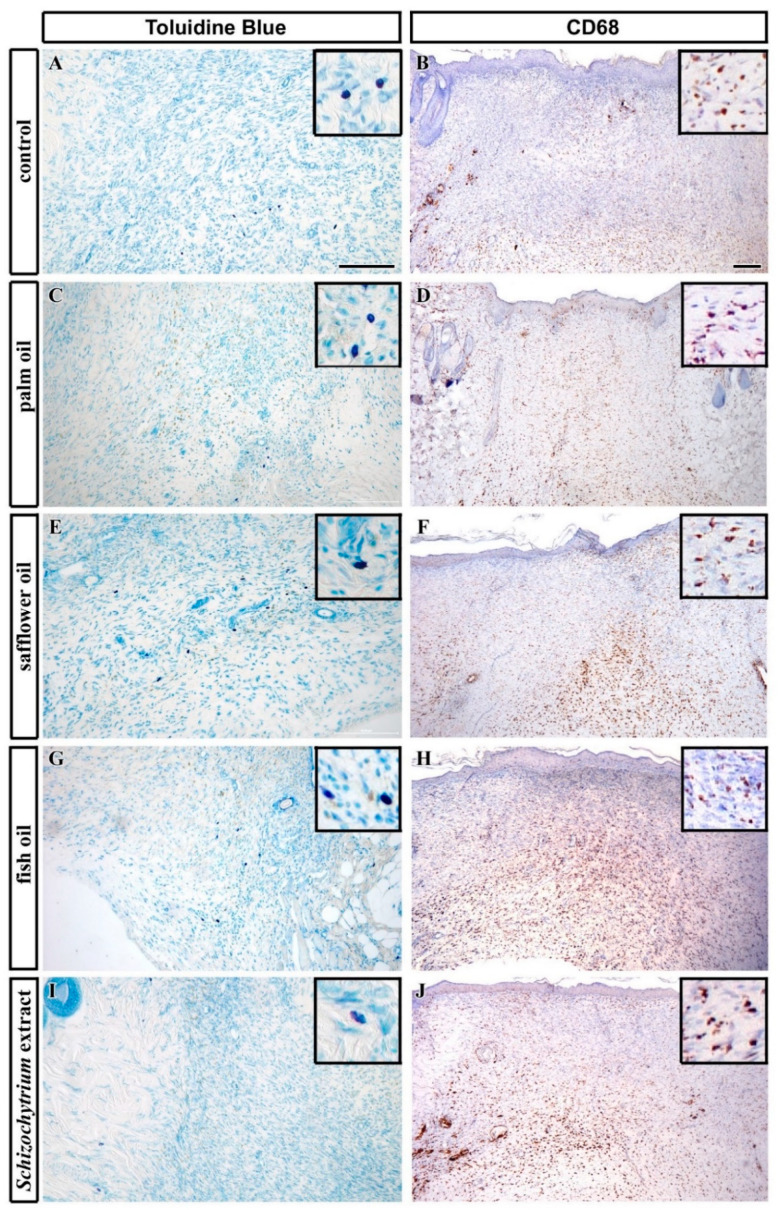
Detection of immune cells in skin of experimental rats 12 days after excision. (**A**,**B**): Control animals, (**C**,**D**) palm oil fed animals, (**E**,**F**) safflower oil, (**G**,**H**) fish oil, and (**I**,**J**) *Schizochytrium* extract treated animals were analyzed 12 days after skin excision. Mast cells labeling by Toluidin Blue staining (blue, photos taken from the middle “B” layer of the dermis; see Figure 14C for the reference) and immunolabeling of macrophages by CD68-positivity (brown, low power view) in transversal sections through the dorsal skin. Inserts display high power images of labeled cells. Scale bar = 100 µm.

Analyses of neutrophils was performed by detection of MPO-positive cells. The number of MPO-positive cells was the lowest in the palm oil diet and the highest in the fish oil-treated group and this difference was statistically significant in comparison to control (Figure 6). Mastocytes were labeled by Toluidine Blue staining and macrophages by CD68 immunolabeling. Furthermore, the number of mast cells was the highest in the fish oil-treated group and the lowest in dietary supplementation with palm oil (Figure 7 and Figure 8), however, because of large variability among samples, these results were not confirmed to be statistically significant. 

Only safflower oil-treatment displayed statistically significant reduction in the number of mast cells when comparing to controls (Figure 7). The exact number of macrophages was difficult to evaluate as a large number of CD68-positive cells was labeled especially in the deeper layers of the skin (Figure 8), therefore only semiquantitative evaluation was performed (Table 1). The amount of macrophages was also the highest in the fish oil-treated animals and very low in palm-oil treated animals (Figure 8, Table 1).

**Figure 9:**
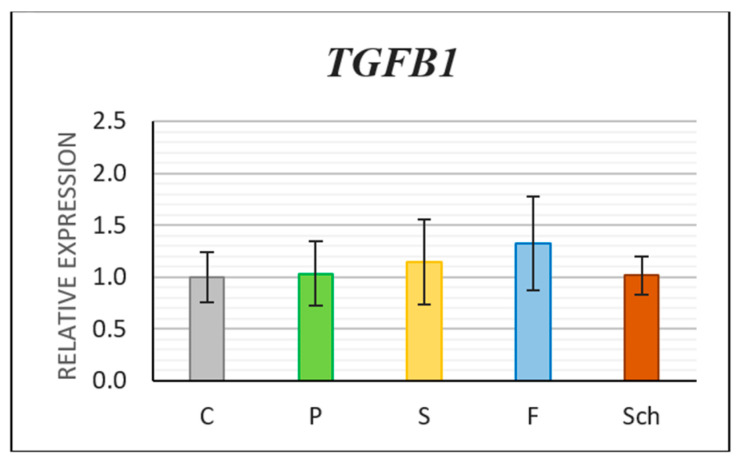
Relative gene expression of Transforming Growth Factor β1 (*TGFB1*). Control (C), palm oil (P), safflower oil (S), fish oil (F), and *Schizochytrium* extract (Sch) treated animals were analyzed 12 days after skin excision. Average values and standard deviations are displayed in the graph.

Transforming Growth Factor-Beta 1 (TGFB1) is secreted by macrophages after inflammatory stimuli; therefore, we selected this marker for further gene expression analyses. Gene expression of *TGFB1* was not significantly changed in any of the experimental diets (Figure 9); however, slight enhancement of expression was observed after fish oil treatment in the healing skin 12 days post-excision in comparison with control. 

### 2.5. Maturation of Collagenous Tissue in Dermis Following the Intake of Oil Supplemented Diet

**Figure 10:**
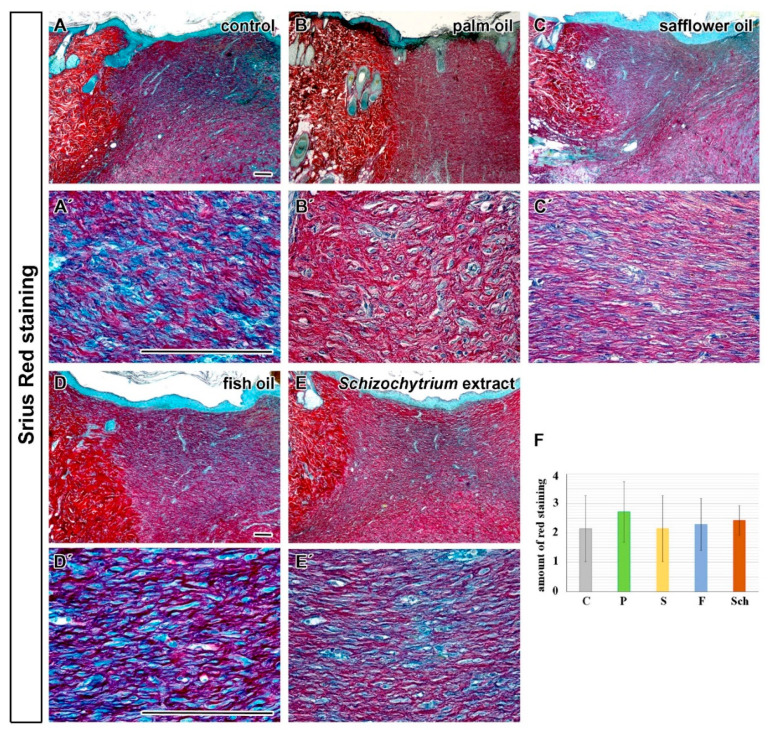
Sirius Red staining to visualize collagen maturation in dermis. (**A**,**A’**): Transversal sections through skin at 12 days post-excision in the samples of the healing skin of rats fed by a standard diet alone (control, C); (**B**,**B’**): a standard diet enriched with palm oil (P); (**C**,**C’**): diet enriched with safflower oit (S); (**D**,**D’**): diet enriched with fish oil (F); (**E**,**E’**): diet enriched with *Schizochytrium* extract (Sch), respectively. (**F**): Quantification of Sirius Red staining. Intensity of staining was evaluated and “+” was labeled as the less mature collagenous tissues (intense purple/blue staining) and “++++” the most mature collagenous tissues (intense red staining), see Figure 14D–D’’’for the explanation of data analyses. Median values and standard deviations are displayed in the graph. Scale bar = 100 µm.

Total collagen maturation was first semi-quantified based on the Sirius Red staining (Figure 10A–F) The intensity of red staining was the highest in the healing skin of rats fed the diet with palm oil (Figure 10B,B’) and *Schizochytrium* extract at 12 days post-excision (Figure 10E,E’) but all treatments exhibited some extent of collagenous tissue maturation in the dermis of wound area (Figure 10).

**Figure 11:**
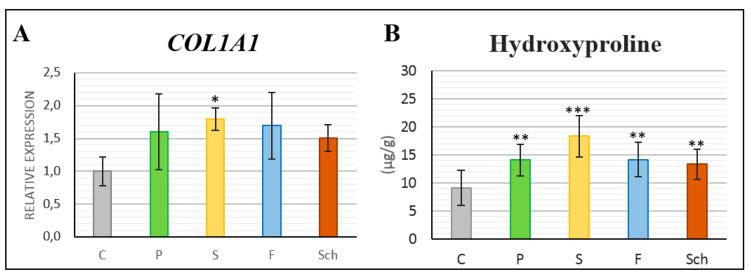
Gene expression of *COL1A1* and hydroxyproline content in wound area. Control (C), palm oil (P), safflower oil (S), fish oil (F), and *Schizochytrium* extract (Sch) treated animals fed for 64 days were analyzed 12 days post-excision. (**A**): *COL1A1* gene expression analyses. * labeled group of fatty acids, which differ significantly from the control group (*p* < 0.05; unpaired *t*-test; *n* = 3). (**B**): Analysis of hydroxyproline content. Stars label group of fatty acids, which differ significantly from the control group (* *p* < 0.05; ** *p* < 0.01; *** *p* < 0.001, unpaired *t*-test; *n* = 10). Average values and standard deviations are displayed in both graph.

Also, on gene expression level, we observed some changes of *COL1A1* level in the skin samples (Figure 11A). All supplementary oils slightly increased *COL1A1* expression, but statistically significant increase was observed only in safflower oil group (*p* < 0.05) in comparison with the controls. 

Next, we analyzed the amount of hydroxyproline in skin samples of animals treated with different diets. The highest increase in hydroxyproline concentration was observed in the healing skin tissue of dietary safflower oil when comparing to control. However, statistically significant difference was observed also in other supplementary diets (Figure 11B).

### 2.6. MALDI-TOF Mass Spectrometry Analysis of Collagen III Fragment Revealed its Decrease in Fish Oil Supplementation

**Figure 12:**
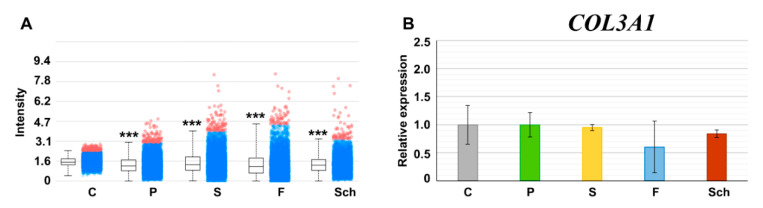
MALDI-TOF mass spectrometry analysis of collagen III fragment and gene expression of *COL3A1*. (**A**): Intensity box plot for the *m*/*z* 1679.873 ± 0.100 Da collagen α-1(III) chain fragment GAPGFRGPAGPNGIPGEK 12 days post-excision in the dermis of rats fed 64 days a standard diet alone (control, C), or a standard diet enriched with palm oil (P), safflower oil (S), fish oil (F), and *Schizochytrium* extract (Sch), respectively. Each dietary group is represented by six tissue sections (*n* = 6) with several tens of thousands of the mass spectra measurements within each section; intensity of a mass peak from each mass spectrum is depicted by a spot within the plot (C > S > Sch > P > F, *** *p* < 0.001, Kruskal–Wallis test). (**B**): Gene expression of *COL3A1* (*p* < 0.05; unpaired *t*-test; *n* = 3). Average values and standard deviations are displayed in the graph.

MALDI-TOF MSI was used to uncover differences in signal intensity of the specific collagen III fragment of amino acid sequence GAPGFRGPAGPNGIPGEK (Figure 12A). Despite relatively small differences between the dietary groups (including a relatively high variability outside the upper and lower quartiles), due to the very high number of measurements of a given fragment within each dietary group, the differences were statistically significant in comparison to controls and an amount of this particular collagen fragment decreased in the healing skin wound 12 days post-excision in the sequence C > S > Sch > P > F.

Moreover, differences in *COL3A1* level were observed between individual diets during gene expression analyses by QPCR (Figure 12B). The lowest level of expression was also observed in fish oil supplementation; however, the difference was not statistically significant in comparison to controls. 

## 3. Discussion

### 3.1. Availability of Polyunsaturated Fatty Acids for Wound Healing

In order to evaluate the availability of EPA/DHA for wound healing, we measured their deposition in the visceral adipose tissue. We selected this tissue as the target organ based on previously published data [24] where fatty acid composition was measured for brain, heart, liver, skeletal muscle, erythrocytes, plasma phospholipids, adipose tissue, and plasma triglycerides using twelve moderate-fat diets in rats. These analyses revealed that the adipose tissue triglycerides were the most responsive of all the tissue lipids to the diet PUFA balance over the full linear range (with a slope of 0.79).

### 3.2. Effect of Polyunsaturated Fatty Acids n-3 on Wound Healing

An effect of oils with high content of n-3 polyunsaturated fatty acids on wound healing was previously described in different experimental conditions, and it was proven to be involved in alteration of wound closure or in improvement of the endothelial function through modulation of cytokines [15,17,18]. Here, we focused on different dietary supplements with variable amount of EPA and DHA as well as PUFA n-6 oils and their possible effect on cellular responses during wound healing in rat dorsal skin. 

Our study determined that safflower oil (high level of PUFA n-6) increased, and dietary supplementation with *Schizochytrium* oil (high level of DHA) decreased, all measured parameters of the wound extent on day 12 post-excision, indicating differential effect of PUFA n-3 and PUFA n-6 diet on the wound closure. Therefore, the process of closure was slower following PUFA n-6 treatment. Similarly, a previous study of dos Santos Rosa et al. [18] revealed differences in the speed of tissue closure as reaction on different oil supplements, where olive oil (monounsaturated fatty acids) inhibited stress-induced reduction in wound contraction and re-epithelialization were conceived in mice 14 days after excisional lesions [18]. On the other hand, fish oil with high level of EPA caused slower closure of wound at macroscopic analyses [18], similar to our analysis measuring superficial width of the wound. Shallower wounds were also observed 5 days after skin penetration in rats receiving parenteral fish oil emulsion [2]. Moreover, Otranto et al. [17] reported slower wound closure and smaller percentage of re-epithelialization in the fish oil group in comparison to control animals. 

Interestingly, *Schizochytrium* oil decreased the wound area as well as other wound parameters when compared to fish oil in the present study, despite the fact that the differences between the Sch and F groups were not statistically significant. Therefore, an ambiguous effect of PUFA n-3 on the wound extent found previously [2,17,18,25] as well as in our experiment, seems to be associated with different content of EPA and DHA in these diets and it will be necessary to follow this in detail in future. Moreover, it is necessary to mention that bioavailability of oils, especially in the case of omega-3, exhibits a unique dynamic for the incorporation in membranes and the balance of n-3 and n-6 PUFA is critical for the membrane composition [26]. As DHA became active after its incorporation in membrane phospholipids, in future experiments it will also be necessary to provide the evidence that DHA is incorporated in membrane phospholipids of target tissues for individual diets.

We should also mention that there are species–specific differences in the type of wound closure, where human and pig tissues heal mostly by re-epithelialization but in rodents, excessive skin contractions occur. These contractions are generated by myofibroblasts in the dermis and they cause the movement of skin edges towards the centre of the wound. A larger wound extent (wound size) can therefore, in rat, be interpreted as the reduction of the wound contraction. While in humans, contraction of wounds is an unwanted side-effect resulting in scarring, a slower reduction in wound size in rodents could be a positive asset.

### 3.3. Angiogenesis and Inflammation Are Affected by Oil Supplementation during Skin Healing

An ability of the tested dietary oils to facilitate the formation of a granulation tissue including its angiogenesis and its inflammatory response was assessed in our study from several aspects. As far as inflammation is concerned, PUFAs n-3, especially DHA, are a source of the anti-inflammatory mediators: resolvins, protectins, and maresins [26]. These molecules are able, among other things, to decrease excessive accumulation of neutrophils at the site of inflammation [9,10]. In this regard, increased numbers of macrophages, mastocytes, and especially neutrophils found out in the healing skin of the fish oil-treated rats in the present experiment do not agree with these data, and it would be useful to measure the above-mentioned mediators in a future experiment. However, because the inflammatory phase is a necessary component of the process of wound healing, McDaniel et al. [15] argued that dietary PUFA n-3 demonstrate ability to affect the local production of inflammatory mediators that regulate the wound healing process, but whether these mediators are beneficial or detrimental is not clear, and how they specifically influence wound healing is still not clearly understood. 

An extent of neo-angiogenesis in rats is the highest between the ninth and fifteenth day after skin penetration [27] in the case that the assessment is based on the evaluation of the alpha-SMA-positive cells. Our experiment (12 days post-excision) was designed to target this period of wound healing. An increased number of vessels were observed following safflower, fish, and *Schizochytrium* extract with the highest number of vessels located in the deep layers in safflower oil-treated animals, while vascularization of superficial layers was highly enhanced in *Schizochytrium* extract treated rats. However, it is important to mention that only larger blood vessels can be visualized by alpha-SMA immunohistochemical labeling but very small vessels consisted of only thin wall with endothelial cells could not be detected this way. The alteration of vessels number corresponded to *VEGFA* gene expression changes. This is in agreement with the findings of Otranto et al. [17], where the volume occupied by blood vessels in rats 14 days after wounding was also increased in the fish and linseed oil group (rich in PUFA n-3) in comparison to controls both in the superficial and deep skin regions. *VEGFA* expression was slightly increased at 14 days after wounding in fish oil treated rats [18]; however, contrary to these findings, the number of vessels was found out to be reduced (despite the fact that superficial and deep layers were not evaluated separately). Similarly, decreased angiogenesis was demonstrated in the skin-wounded rats following intra-peritoneally administered fish oil emulsion [2]. However, an earlier time point was evaluated in this study (5 days after skin excision) and the exact number of vessels was not evaluated; just semi-quantitative analyses were performed. Based on our findings, it is critical to well define evaluated areas and analyze superficial and deep areas for angiogenesis separately as it can be a reason for discrepancies found in literature.

TGFB1 regulates multiple cellular processes in all major phases of cutaneous wound healing [28]. During the proliferative phase studied by us (day 12 post-excision), it is produced by macrophages and contributes to the formation of granulation tissue by promoting angiogenesis (stimulation of endothelial cell migration), increasing fibroblast proliferation, promoting fibroblast trans-differentiation into myofibroblasts, or stimulating extracellular matrix production [28]. Up-regulation of *TGFB1* on the RNA level was previously associated with increased formation of granulation tissue, the enhanced neo-vascularization as well as higher collagen content on day 15 post-wounding in a rat model [29]. We have not observed significant differences in *TGFB1* expression in our study and we were not able to confirm these findings; however, fish oil tended to increase *TGFB1* expression in comparison with other dietary interventions, the fact corresponding to increased inflammatory cells distribution and increased *VEGFA* expression associated with increased angiogenesis. On the other hand, decreased mean scores were reported for the epidermal and dermal regeneration and granulation tissue thickness in rats by intra-peritoneal injections of fish oil emulsion for 5 days after dorsal skin wounding [2]. However, it is necessary to mention that our experiment targeted only one time point of tissue healing (12 days) and therefore early phases of tissue response to dietary oil supplementation were not evaluated and their enhanced effect, especially on mRNA level, could occur during the initiation phases of tissue healing. 

### 3.4. Dermis Healing and Collagenous Tissue Maturation

Approximately one-quarter of all amino acid residues in mammalian collagens are L-proline, of which nearly 40% is post-translationally converted to L-hydroxyproline [30]. Therefore, the standard procedure for an estimation of the extent of collagen production is based on the determination of hydroxyproline [1]. The measurement of hydroxyproline can also be used as a surrogate for collagen quantity in the neo-dermis [31]. The highest increase in hydroxyproline concentration was in our experiment observed in the healing skin tissue of all dietary oils in comparison to control with similar trends observed in *COL1A1* expression analysis where safflower oil caused the most significant upregulation. In agreement, Sirius Red staining uncovered less intense red staining, demonstrating the lower maturation of collagenous tissues, in safflower oil-treated group. Similar to our results, higher content of hydroxyproline was detected 14 days post-excision in the healing skin of rats treated by fish oil and linseed oil [17]. In contrary, another study found lower level of hydroxyproline production following fish oil treatment [18] also 14 days post-excision. It is essential to mention that hydroxyproline is usually quantified spectrophotometrically [18], but a more accurate HPLC method can be used, as was preferred also by us. These different analytical approaches could affect above reported results and may be the basis of found discrepancies. 

Moreover, collagen III is essential for a proper skin function and its higher proportion improves the healing; however, as the healing process progresses, collagen III is replaced by collagen I to increase the tensile strength [32]. A substantial replacement of collagen III by a definitive collagen I was expected in our experiment, as it is typical for this phase of the granulation tissue production (12 days post-excision) as was reported previously [33]. Collagen III was found to be present in a significant amount in all dietary groups, with the lowest amount in the fish oil treated group and the highest in the safflower oil treated group. The ratio of collagen I/III is 4/1 in a healthy skin. From this viewpoint, safflower oil seems to accelerate the healing with collagenous tissue maturation as the last one from all analyzed treatments at this late time point. This finding was also supported by high *COL1A1* gene expression and the largest wound extent in safflower oil treated animals. 

In the case of the *Schizochytrium* extract treated group, papillary dermis also contained collagen I. Therefore, an even distribution of collagen III and collagen I in the healing wounds of *Schizochytrium* extract treated animals together with a smaller extent of wound without induction of augmented immune cell response indicate a more advanced steps of healing process in these animals in comparison with other experimental groups.

The lowest expression of *COL3A1* was found in the healing skin of rats fed by the fish oil with high inflammation in granulation tissue, which corresponded with the data obtained by the MALDI MSI (the lowest content of the collagen III fragment in the fish oil treated group from all tested dietary interventions). MALDI MSI, a technique for spatially resolved molecular analysis of tissue sections, has a high potential to complement and support histological analysis [34,35]. As far as wound healing is concerned, MALDI MSI was, until now, used only in the in vitro skin model for the evaluation of the lipid distribution in the cosmetics study [36]. We used this technique previously in the rat model of cutaneous wound healing [37] and found higher content of a collagen III fragment in the fish oil-fed rats than in the present study. One likely reason of this difference is the use of cryosections in the present study and formalin-fixed-paraffin-embedded tissue sections in the previous study [37]. Paraffin embedded tissues needs to undergo deparaffinization and antigen retrieval, during which the analytes of interest can be diluted and delocalized in the tissue, with a consequence of the worse efficiency of their ionization in comparison to cryosections [38]. This disadvantage should be taken into account during analyses.

## 4. Materials and Methods

### 4.1. Chemicals

All chemicals were purchased from Sigma-Aldrich (St. Louis, MO, USA) in ACS purity, unless mentioned otherwise.

### 4.2. Animals, Dietary Interventions

Fifty adult male rats of the laboratory strain Wistar Albino (Bio Test, Konárovice, Czech Republic) at the age of 8 weeks (the mean live weight of 225 ± 21.3 g) were used. The rats were housed in the plastic boxes (53.5 × 32.5 × 30.5 cm) of four animals each in a room maintained at 23 ± 1 °C, humidity of 60% and 12/12 h of light/dark cycle (maximum intensity of 200 lx). The experiment was performed in a compliance with the Czech National Council Act No. 246/1992 Coll. to protect animals against cruelty, the amended Act No. 162/1993 Coll., and was approved by the “Commission to protect animals against cruelty” of the Mendel University in Brno (Statement No. 16252-MZE-17214 18. 4. 2018). 

The rats were divided into five groups by 10 animals each and fed for a total of 64 days either the control (C) basic feed mixture (pelletized complete chow for mice and rats; Biokron, Blučina, Czech Republic) or the basic feed mixture supplemented with 8% of fish oil (F), 8% of oil extracted from the *Schizochytrium* microalga (Sch), 8% of safflower oil (S), and 8% of palm oil (P), respectively. Fatty acid composition in all five used diets is shown in Table 2.

The diets were prepared (at the laboratory) as follows: pelletized chow was ground, homogenized with an appropriate amount of a particular oil, and the cakes weighing approximately 200 g were prepared by hand. The animals were fed daily ad libitum with free access to drinking water. Feed consumption was measured daily per a given box; daily feed consumption per rat was calculated as a one-fourth of the total intake per cage (due to the ad libitum access to the feed, significant differences in consumption between animals in a box were not expected) and animals were weighed in weekly intervals.

### 4.3. Excisions, Sample Collection

**Figure 13:**
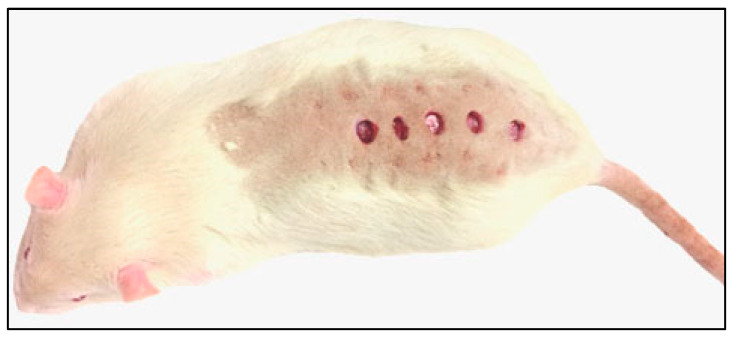
Localization of individual excisions on rat dorsum.

After 52 days of fattening, all animals were anesthetized by the intramuscular application of zolazepam (Virbac, Carros, France), and five full-thickness (epidermis, dermis) cutaneous excisions on dorsum in a sagittal plane were performed using a circular puncher with an internal diameter of 8 mm (Stiefel Laboratories, Brentford, UK; Figure 13). 

After excision, the rats were housed individually: two animals were placed in the same plastic box as mentioned above, partitioned with a crossbar into two parts. After 12 days of continuation on the particular diet, the animals were sacrificed by isoflurane overdosing and the following tissues were taken: five samples of the healing skin for gene expression, hydroxyproline determination, production of the histological preparations, immunohistochemistry (IHC) preparations and matrix-assisted-laser-desorption-ionization time-of-flight mass-spectrometry-imaging (MALDI-TOF MSI), respectively, and the 5 g aliquot of the visceral adipose tissue (VAT) for the determination of fatty acid deposition.

### 4.4. Determination of Fatty Acids Content in Individual Diets

Fatty acid (FA) content was measured in individual diets and their deposition was quantified in the visceral adipose tissue as the most responsive metabolic organ to the diet with PUFA balance [24] in order to evaluate FA availability for the healing process. Fatty acid content was determined according to our previous study [39] with the following exceptions: fatty acid methyl esters (FAMEs) were separated using a Fisons GC 8000 series chromatograph (Fisons, Ipswich, UK) on a DB-23 capillary column with dimensions of 60 m × 0.25 mm and a particle size of 0.25 µm (Agilent Technologies, J&W Scientific, Santa Clara, CA, USA); the GLC -455 (Nu-Chek-Prep, Elysian, MN, USA) was used as an external standard for FAMEs identification. FA content was expressed in the diets as a percentage of the sum of all determined fatty acids and in g/100 g of the fresh tissue, respectively.

### 4.5. Quantification of Gene Expressions

Total RNA was isolated from the cutaneous healing tissue (30 mg) using RNeasy Lipid Tissue Mini Kit (Qiagen, Valencia, CA, USA). The quality of isolation was checked on the 1.2% RNA gel visualized by ethidium bromide. The concentration of isolated RNA was measured on spectrophotometer NanoDrop 2000 (Thermo Scientific, Waltham, MA, USA). Isolated RNA was stored at −80 °C. One microgram of the isolated RNA was reverse transcribed using Omniscript RT Kit (Qiagen, Valencia, CA, USA) and oligo-dT primers. TaqMan Gene Expression Assay (cat. No. 4331182, Applied Biosystems, USA) for the rat *Transforming Growth Factor β1* (*TGFB1*; Assay ID: Rn00572010_m1); *Vascular Endothelial Growth Factor* (*VEGF*; Assay ID: Rn01511602_m1); *Collagen 1 Alpha 1* (*COL1A1*; Assay ID: Rn01463848_m1); *Collagen 3 Alpha 1* (*COL3A1*; Assay ID: Rn01437681_m1); *Beta-Actin* (reference gene *ACTB*; Assay ID: Rn00667869_m1). 

qRT-PCR was analyzed with a LightCycler^®^ 480 (Roche, Basel, Switzerland) with the following program: initial activation step at 95 °C for 10 min, followed by 40 cycles at 95 °C for 15 s, annealing temperature at 62 °C for 60 s.

The differences between expression of the gene of interest (GOI) in the sample (P, S, F, and Sch, respectively) and in the control (C) were calculated using the ΔΔCT method according to Schmittgen and Livak [40] based on the determined effectivity (E) and the quantification cycle value CT for the GOI and the reference gene (REF) as (1+E)^−{[CTGOI(sample) − CTREF(sample)] − [CTGOI(control) − CTREF(control)]}^.

### 4.6. Determination of Hydroxyproline Amount

Hydroxyproline was separated on the column Zorbax Eclipse AAA (150 × 4.6 mm, particle size of 3.5 µm; Agilent Technologies, Santa Clara, CA, USA) using high performance liquid chromatograph HP 1100 Series (HP, Palo Alto, CA, USA). The column was thermostated at 40 °C. Mobile phase A consisted of 40 mM Na_2_HPO_4_ at pH 7.8 (5.5 g of Na_2_HPO_4_ monohydrate/1 l of H_2_O, adjusted to pH 7.8 with 10 M NaOH) and mobile phase B was acetonitrile/methanol/water (45:45:10 *v*/*v*). Flow rate of the mobile phase was 2 mL/min. The compounds were eluted with a linear upward gradient 0% B 0.0 min → 57% B 0.8 min → 100% B 10.0 min → 0% B 12.5 min to 14.0 min. The column effluent was monitored with a fluorescence detector at 340ex/450em nm using ortho-phthaldialdehyde as a precolumn derivatization agent. The HPLC chromatographic system was controlled with ChemStation software (rev. A 07.01).

### 4.7. Assessment of the Wound Extent 

Tissue samples of the healing skin were fixed in 10% buffered formalin, dehydrated by a gradual alcohol series, cleared in xylene, embedded in paraffin blocks and sectioned into the size of 8 µm using a rotary microtome Leica RM2145 (Leica Microsystems, Wetzlar, Germany). Sections were subsequently stained with Hematoxylin-Eosin (HE; evaluation of the wound extent) and Masson’s Green Trichrome (MGT; assessment of the total collagen maturation), respectively.

**Figure 14:**
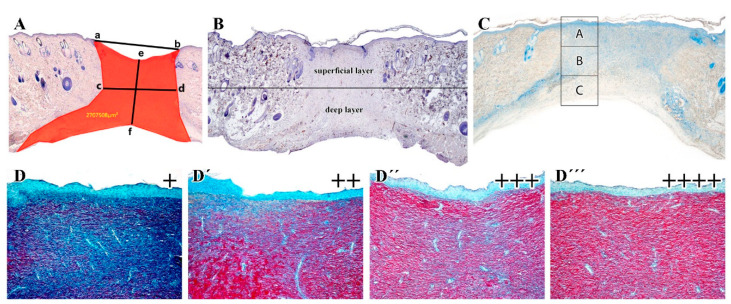
Overview of methodological approaches used for quantification of healing parameters. (**A**): Wound extent parameters: total wound area (red), superficial width (a–b), middle width (c–d), depth (e–f). (**B**): Localization of the borderline between the superficial and deeper layers of the dermis. (**C**): Quantification of Toluidine Blue-positive cells was performed in three distinct areas: superficial (A), middle (B) and deep (C) dermis (**D**–**D’’’**): Semi-quantification of collagen tissue maturation was performed on Sirius Red stained sections.

HE and MGT samples were examined using a Leica DM LB2 Video Microscope (Leica, Wetzlar, Germany). At least 10 microscopic fields from each group were digitized then assessed by Keyence software version 1.04 (Keyence, Mechelen, Belgium); the values of the wound depth based on the HE-staining were expressed as a percentage of the surrounding unwounded skin. The wound extents were evaluated by analysis of following parameters (Figure 14A): total wound area (red area), wound width in the superficial area (a–b), wound width in the deep area (c–d), and in the wound depth (e–f), respectively. 

It is necessary to mention that measurements of wound width are difficult to make when circular wounds are used. Circular wounds have a sixfold greater variance, since the circular geometry of punch wounds makes it technically difficult to take a microscopic section from the middle of the wound or perpendicular to the wound edge [41]. For this reason, we cut all samples through whole wound and then analyzed only the most central (the largest) areas of the wounds. 

### 4.8. Immunohistochemistry of Immune Cells and Special Staining of Collagen Fibers 

After deparaffinization and rehydration of the sections, citrate buffer (pH = 6) was used as a pre-treatment in a 98 °C water bath. For inhibition of non-specific secondary antibody binding, sections were incubated with a blocking serum (VECTASTAIN ABC Kit, Rabbit IgG, PK-4001, Vector Laboratories, Burlingame, CA, USA) for at least 20 min at room temperature (RT) and then, they were incubated with the primary antibody (alpha-SMA, CD68, MPO; detailed information Table 3). 

After application of biotinylated secondary antibody (VECTASTAIN ABC Kit, Rabbit IgG, PK-4001, Vector Laboratories, Burlingame, CA, USA) for 30 min at RT, slices were incubated with the peroxidase-conjugated avidin-biotin complex (VECTASTAIN ABC Kit, Rabbit IgG, PK-4001, Vector Laboratories, Burlingame, CA, USA) for 30 min at RT. Chromogen substrate diaminobenzidine (Liquid DAB+ Substrate Chromogen System, K3468, Agilent Dako, Santa Clara, CA, USA) was used for visualization of positive cells. Counterstaining of slices was performed by Hematoxylin. A negative control of the sample was obtained by omitting the primary antibody from the labeling protocol.

Photos were taken by light microscope (Leica DM5000 B, Leica Microsystem GmbH, Vienna, Austria) and a digital color camera (Leica DFC480, Leica Microsystem GmbH, Vienna, Austria).

Level of angiogenesis was semi-quantified on immunohistochemically labeled sections, where vessels were recognized by the presence of smooth muscle cells expressing alpha-smooth muscle actin (alpha-SMA) in the vessel wall. The number of vessels was counted on the entire wound area in each sample (at least 10 microscopic fields from each group). Moreover, the number of vessels was also determined separately in the superficial and the deeper layer of the dermis, respectively. The borderline between the layers was set in the middle of wound depth (Figure 14B) where the composition and arrangement of collagen fibres modified.

The semi-quantitative analysis of total collagenous tissue maturation (using the Sirius Red staining) was carried out according to Maia-Figueiro et al. [42] with a slight modification. The slides were classified in four grades (I-IV) according to the red intensity: grade I (+), grade II (++), grade III (+++), and the reddest as grade IV (++++). Two sections were analyzed from 4 animals of each treatment (Figure 14D–D’’’).

Detection of macrophages was performed by immunolabeling of CD68-positive cells (Table 3) on 2 sections from 4 animals of each treatment. Analysis was performed separately on the superficial and deep layer (Figure 14B). The slides were classified in four grades (I-IV) according to the amount of CD68-positive cells: grade I (+), grade II (++), grade III (+++) and the highest presence of positive cells as grade IV (++++).

Neutrophils were visualized by the detection of MPO-positive cells (Table 3) on 2 sections from 4–5 animals of each treatment on the wound margin (Figure 14C). Toluidine Blue was used to detect mastocytes and analyses were also performed on 2 sections from 5 animals for each treatment on the wound margin (Figure 14C). MPO-positive cells and Toluidine Blue-positive cells were counted in three areas (A: superficial, B: middle, and C: deep layer) of the dermis to see possible differences in their distribution through the tissue. 

### 4.9. MALDI-TOF MSI

Cryosections with 10 µm thickness were mounted onto ITO (indium-tin oxide) glass slides and for the MALDI MSI measurements were prepared according to Heger et al. [43]. The mass spectrometry imaging was performed on a MALDI-TOF/TOF mass spectrometer Bruker ultrafleXtreme (Bruker Daltonik GmbH, Bremen, Germany) using a protocol according to Heger et al. [43]. A total sample set spanned 10 ITO glass slides containing 30 tissue sections. Six tissue sections per dietary group were used. As MALDI matrix was used 30 mg/mL 2,5 dihydroxybenzoic acid (DHB) in methanol/water (50:50, *v*/*v*) with 1% trifluoroacetic acid (TFA). Raster spot diameter was 30 μm. MALDI MSI of peptides was performed in reflector positive mode in the *m*/*z* range 0–5 kDa. A total of 500 spectra were summed for each spot using the Random Walk raster pattern. The MSI data were processed using SCiLS Lab 2014b software (SCiLS – Bruker Daltonik GmbH, Bremen, Germany).

Collagenase A from *Clostridium histolyticum* was used for *on-tissue* digestion. Approximately 300 μL of collagenase A solution (1 mg/mL in water) per one slide was applied by ImagePrep (Bruker Daltonik GmbH, Bremen, Germany), the slide was kept inside small box with humid atmosphere for 24 h at 37 °C, and then the DHB matrix solution was applied. The slide was dried in vacuum desiccator for 15 min before MSI analysis.

### 4.10. Statistical Analysis 

MSI data were evaluated in SCiLS Lab software according to Guran et al. [35] using the Anderson–Darling normality test and the Kruskal–Wallis test. Regarding the other sets of data, the differences between dietary groups were evaluated by unpaired *t*-test and they were considered significant at the level of *p* < 0.05. The STATISTICA 12 package (StatSoft, Tulsa, OK, USA) was used for these evaluations. 

## 5. Conclusions

Our study confirmed that wound healing processes in skin are modulated by the content of supplementary oils in diets. Dietary oil containing high PUFA n-6 caused delay of healing processes including wound closure and enhanced angiogenesis. The unequal effect of dietary oils rich in PUFA n-3 (fish oil and *Schizochytrium* extract) revealed by our experiment could be explained by different level of EPA/DHA in these oils and it will need to be followed in future as the high level of EPA or DHA seems to be driving differential tissue response during skin healing. Fish oil (high EPA) induced high immune cell response, while *Schizochytrium* extract (high DHA) enhanced wound closure without elevated inflammatory reaction. Thus, different dietary supplementation can specifically enhanced individual staged of wound healing, which should be taken in account in future studies.

## Figures and Tables

**Table 1:** Semi-quantitative analyses of macrophages by immunolabeling of CD68-positivity.

	Superficial Layer				Deep Layer			
	Sample 1	Sample 2	Sample 3	Sample 4	Sample 1	Sample 2	Sample 3	Sample 4
control	++	+++	++	++	+++	++++	++	++
palm oil	++	++	++	+	+++	+++	+++	++
safflower oil	++++	+++	++++	++	+++	+++	++	+++
fish oil	++	++++	+++	++	+++	+++	++++	++
*Schizochytrium* extract	++	++	+++	+++	+++	+++	+++	++

Control (C), palm oil (P), safflower oil (S), fish oil (F), and *Schizochytrium* extract (Sch) treated animals were analyzed 12 days after skin excision. All analyzed samples are displayed to demonstrate variability among animals. The slides were classified into four grades (I–IV) with the lowest amounts of CD68-positive cells labeled as grade I (+) and the highest presence of CD68-positive cells labeled as grade IV (++++).

**Table 2:** Content of fatty acids in the standard rat diet (control, C) and standard diets enriched with 8% of palm oil (P), safflower oil (S), fish oil (F), and *Schizochytrium* extract (Sch), respectively.

Fatty Acid	Diet (% of the Sum of Total Fatty Acids)				
	C	P	S	F	Sch
14:0	0.3	0.9	0.4	0.1	3.7
16:0	23.4	42.0	22.5	19.4	15.7
17:0	0.2	0.2	0.3	0.6	0.2
18:0	4.7	5.3	6.5	3.7	2.0
16:1n-7	0.5	0.2	0.5	8.3	0.3
18:1n-9	56.2	44.9	48.6	33.4	25.0
18:2n-6	9.4	2.2	17.3	2.0	2.3
18:3n-6	4.1	3.2	2.5	4.5	1.3
20:2n-6	0.1	0.0	0.1	0.4	0.1
20:3n-6	0.0	0.0	0.0	0.1	0.3
20:4n-6	0.1	0.1	0.2	0.3	0.5
22:4n-6	0.1	0.1	0.1	0.2	1.0
18:3n-3	0.1	0.1	0.0	0.3	3.3
20:5n-3	0.1	0.1	0.3	8.7	0.9
22:5n-3	0.5	0.2	0.5	1.3	7.4
22:6n-3	0.2	0.5	0.2	16.7	36.0

**Table 3:** List of antibodies used for immunohistochemical analysis.

Primary Antibody	Company	Catalog No.	Host Species	Dilution	Time/Temperature
alpha-SMA	Abcam, London, UK	ab5694	rabbit	1:100	60 min/RT
MPO	Abcam, London, UK	ab9535	rabbit	1:50	60 min/RT
CD68	Invitrogen, Carlsbad, CA, USA	PA5-81594	rabbit	1:1000	75 min/RT

## Supplementary Materials

The following are available online at https://www.mdpi.com/1422-0067/21/21/7911/s1, Figure S1: Fatty acid content in individual diets; Figure S2: Weight characteristics in experimental animals.
