# Supplementary material for: Comparison of Dietary Oils with Different Polyunsaturated Fatty Acid n-3 and n-6 Content in the Rat Model of Cutaneous Wound Healing
Source: Int J Mol Sci. 2020 Oct 24;21(21):7911. doi: 10.3390/ijms21217911 (PMC7672592; doi:10.3390/ijms21217911)
Supplement: Supplementary file 1 [file ijms-21-07911-s001.pdf]

International Journal of *Molecular Sciences*

Article

Comparison of dietary oils with different polyunsaturated fatty acid n-3 and n-6 content in the rat model of cutaneous wound healing

Tomas Komprda[1], Zbysek Sladek[2], Zuzana Sevcikova[3], Veronika Svehlova[1], Jan Wijacki[2], Roman Guran[4,5], Tomas Do[4], Zuzana Lackova[4,5], Hana Polanska[6], Lucie Vrlikova[3], Vendula Popelkova[1], Petr Michalek[4,5], Ondrej Zitka[4,5], Marcela Buchtova[3]*

1 Department of Food Technology, Mendel University in Brno, Zemedelska 1, 613 00 Brno, Czech Republic;
2 Department of Animal Morphology, Physiology and Genetics, Mendel University in Brno, Zemedelska 1, 613 00 Brno, Czech Republic;
3 Laboratory of Molecular Morphogenesis, Institute of Animal Physiology and Genetics, v.v.i., Czech Academy of Sciences, Brno, Veveri 97, 602 00 Brno, Czech Republic
4 Department of Chemistry and Biochemistry, Mendel University in Brno, Zemedelska 1, 613 00 Brno, Czech Republic;
5 Central European Institute of Technology, Brno University of Technology, Purkynova 123, 612 00 Brno, Czech Republic;
6 Department of Pathological Physiology, Faculty of Medicine, Masaryk University, Kamenice 5, 625 00 Brno, Czech Republic
7 Section of Animal Physiology and Immunology, Department of Experimental Biology, Faculty of Science, Masaryk University, Brno 625 00, Czech Republic
* Correspondence: buchtova@iach.cz

Received: date; Accepted: date; Published: date

SUPPLEMENTARY FILES

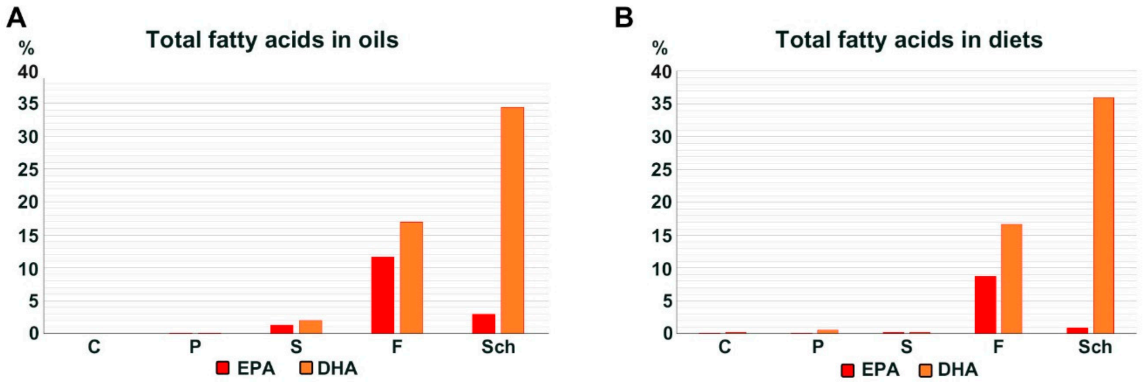

Figure S1. Fatty acid content in individual diets
A, B: Total fatty acids content (EPA in red, DHA in orange) in used oils and individual diets: a standard diet alone (control, C), or a standard diet enriched with 8% of palm oil (P), safflower oil (S), fish oil (F) and *Schizochytrium* extract (Sch).

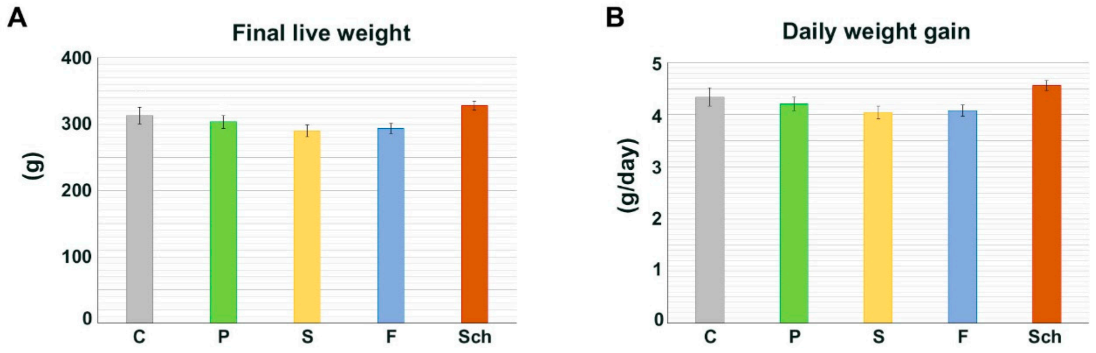

Figure S2: Weight characteristics in experimental animals
A: Final live weight of experimental animals. **B:** Daily weight gain of rats fed 64 days. SFA – saturated fatty acids, MUFA – monounsaturated fatty acids, PUFA – polyunsaturated fatty acids; stars labeled group of fatty acids differ significantly from the control group (* $p<0.05$; ** $p<0.01$, *** $p<0.001$, unpaired t-test; $n = 10$).
